# Research using hashtags: A meta-synthesis

**DOI:** 10.3389/fsoc.2022.1081603

**Published:** 2022-11-25

**Authors:** Gevisa La Rocca, Giovanni Boccia Artieri

**Affiliations:** ^1^Faculty of Human and Social Sciences, Kore University of Enna, Enna, Italy; ^2^Department of Communication Sciences, Humanities and International Studies, University of Urbino Carlo Bo, Urbino, Italy

**Keywords:** hashtag research, hashtag studies, social media, meta-study, meta-synthesis

## Abstract

In the last 20 years, research using hashtags has grown considerably. The changes that occurred in the digital environment have influenced their diffusion and development. Today, there is considerable research on hashtags, their use, and on hashtag activism. Likewise, there is a growing interest in their descriptive measures and their metrics. This article aimed to provide a review of this area of research and studies to outline the traits of hashtag research, which are yet nascent. To achieve this, we used a meta-study to produce a meta-synthesis capable of bringing out similarities and differences in research using hashtags and identifying spaces for the generation of new knowledge.

## Introduction

How are hashtags used in social research? In which direction is the research using hashtags going? This article focuses on these questions, given that hashtag-driven research has grown significantly in recent years and in many fields.

Hashtags, as a research tool, are used in communication (e.g., Bruns and Burgess, 2015), psychology (e.g., Reavley and Pilkington, 2014), sociology (e.g., Bruns et al., 2012), political science (e.g., Lynn et al., 2020), computational social sciences (e.g., Grčar et al., 2017), as well as in the medical sciences (e.g., Tavoschi et al., 2020) and engineering (e.g., Das and Dutta, 2021). Hashtags are used to analyse topic-specific public debates that develop on social media. The research conducted using this tool is also indebted for the technical developments of the features of digital platforms (Helmond, 2015; Gillespie, 2018; van Dijck et al., 2018; van Dijck, 2021) that allow you to scratch data and store information such as, for example, who initiated the conversation and through which hashtag. The hashtag was developed from the programming language C and was initially written as two separate words, hash and tag. However, from the time it was first known as a number sign, the pound symbol, or a tic-tac-toe board, its recently identification as the “hashtag” has changed language and communication for millions (Pandell, 2017). Though merely a label (tag) preceded by the hash sign (#), the hashtag has become more than just a tool for classifying the content of texts and images conveyed through the Internet and through social media. Its meaning, use, and relationship within the social context have undergone a transformation, evolving from a thematic collector—therefore a label that collects all the posts that feed the discussion—to an “ubiquitous sign” (Burgess and Baym, 2020), which produces consequences and effects inside and outside digital environments.

The evolution of the uses of hashtags can be classified based on different periods. In 2007 Chris Messina launched the hashtag within Twitter—a social media platform containing short texts and images—asking users “how do you feel about using # (pound) for groups” (Piatek, 2021). Messina's post was not very successful. The same year, numerous fires broke out in San Diego in October; Natan Ritter—a web developer, who was traveling to San Diego and sighted the flames—started using #sandiegofire to monitor news sources for any information regarding it and then rapidly (every two-to-three minutes) posting information about the fire, road closures and neighbourhood's evacuations on Twitter. In this way, the hashtag helped citizens follow the information and not to disperse it by activating a secondary information channel compared to TV ads (Weller et al., 2014) and to keep constantly updated about the evolution of the crisis. This event demonstrated the usefulness of hashtags for gathering information and creating a sense of community around an event.

In the same period, Vander Wal (2005, 2007) applied to hashtags the concept of folksonomy, a form of cataloging produced from users spontaneously and from below. That is, whereas taxonomies are hierarchical in their classification, folksonomies are created by users within the web to classify events and, at the same time, give them meaning. Folksonomies define situations in which members of a society create words and categories to describe the world in a way that is relevant to them (Neal, 2007). The folksonomies are a result of the users' ability to alter and modify the structure of the conversations on social media from their own words and concepts, without restrictions to terms previously used or predefined by the systems.

With users, hashtags have begun to take on specific uses that are no longer limited to the simple cataloging of events; indeed, it has transformed into a means of associating one's feelings with something that is igniting and concerns the community.

The changes in the usage and what users do with the hashtag has led to research on the changeability of the meanings of hashtags. Colleoni (2013) described them as empty signifiers that invite the ideological identification of a polysemic orientation; that is, it is like an empty jar with a coffee label, and in it, everyone puts their favorite type and brand of coffee. Papacharissi (2016) perfected this definition by considering them as signifiers that are open to definition, redefinition, and re-appropriation by people on social media. The author introduces the possibility of there being different interpretations of what coffee is, of how people can use it and what a generic coffee jar can contain. Hashtags also signify the emotional component users attach to events and affections (boyd, 2010), by expressing participation through expression of sentiment (Papacharissi, 2016). As Bernard (2019) summarizes, the hashtag is a lingua franca that, starting from the function of thematic aggregator (as in the case of the San Diego fires) develops a network of collateral meanings (La Rocca, 2020) produced by those who use them and insert their opinions, feelings, and point of views on specific events.

The study of what is linked to a hashtag, therefore, is of interest to social scientists because it can offer material that allows them to, for example, analyse public debates or reconstruct people's perception of political, social and economic events.

Therefore, the increasing use of hashtags in research can be attributed to the ability of hashtags to share information—words, images, links, and more—hooking them to a continuous conversational flow and offering an observation point to those who want to analyse the evolution and perception of social topics. There are also considerable economic advantages in working with hashtags because they already aggregate the information; additionally, by extracting data through a computer, it is possible to extract them in a short time.

Hashtags have become an important development tool for social research and are now used in various fields. It should, therefore, be possible to reflect on how a hashtag or a chain of hashtags –several hashtags placed in sequence within a post—are used within social research to identify the traits of the nascent hashtag research. Based on these assumptions, we aim to answer the following research question:

[RQ] Through a literature review, is it possible to outline the traits of the nascent hashtag research?

To this end, we resort to a meta-study, specifically looking at the possibilities offered by meta-synthesis.

## A reconstruction of research using hashtags

The aim of this study is to outline the traits of the nascent hashtag research through a review of studies that use hashtags for data collection and interpretation. The object of study is neither digital methods in research nor their possible alternatives (e.g., Marres and Gerlitz, 2016). We also did not focus on the algorithms or hashtag linguistic functions (e.g., Zappavigna, 2015). Our work tries to reconstruct how sector studies use hashtags in research and how they theorize them in these studies. We are trying to connect the different perspectives of using hashtags within social research, to examine similarities and differences, and if possible, at the end of this interpretative process try to find a framework for hashtags that can define the trends of current research. To do this we resort to the meta-synthesis approach by changing the application scenario, since has been used to analyse qualitative research. We resort to it because our intent is to arrive at the interpretation of a nascent, expanding and above all constantly changing phenomenon given the rapid evolution of technologies and meta-synthesis seems to be the most suitable approach to describe and explain the nuances, the interpretative, the paths followed by researchers, having as our ultimate goal to bring out new insights. Our purpose is producing a meta-synthesis attempt to integrate results from a number of different but interrelated studies (Walsh and Downe, 2004). Ours is therefore research on research, which implies not only the analysis of primary research results but incorporates reflection on the perspectives and processes involved in those studies (Clarke et al., 2015). As Zimmer (2006) recalls, for Paterson et al. (2001) what is referred to generically as qualitative meta-synthesis is better designated as the meta-study of qualitative research. In Paterson et al. (2001) the meta-synthesis would be the last of six steps, where the first two “laying the groundwork” and “retrieval and assessment of primary research” represent organizational moments while from the third to the fifth we have “the three analytic steps of meta-study” (p. 109), that is: “meta-data analysis”, “meta-method”, and “metatheory”, concludes this process the “meta-synthesis” (p. 109) follows inductively from these three analytic steps through a “dynamic and iterative process of thinking, interpreting, creating, theorizing, and reflecting” (p. 112).

Ours is a synthesis of studies by different investigators in a related field. Within these studies we try to trace readjusting them from Paterson et al. (2001): the meta-theory, therefore the aspects related to the theoretical perspectives and the social media we are dealing with; the meta-method that requires to examine whether it is a theoretical or empirical study, the methodologies and methods adopted; meta-data analysis, which involves an analysis of the results or conclusions that the study reaches. For the identification of the pertinent documents we followed the indications of Barroso et al. (2003) and the research method called “berrypicking model” (Bates, 1989), so we recovered all the relevant studies in a field, not simply a sample of them, through a non-linear search method but starting from a theme broad “the hashtags” and gradually selecting the articles, the chapters that are most interesting for us and thus refining the terms of the search query. This allowed us to identify a priori two main flows: (1) hashtag studies and hashtag activism; and (2) studies that define hashtag indicators or metrics.

### Appraisal of the first main stream

Scholars in the first stream consider the use and function of hashtags. This first stream of studies builds on and expands the concept of folksonomies that is the possibility for social media users to attribute meanings to events from below through hashtags. These are studies that focus mainly on Twitter because it is the first social media to introduce hashtags. Therefore, starting from these assumptions, two main sub-branches develop: the first is based on the concept of affordances, which refers to the properties of the environment that activate or offer potential action by an agent (Gibson, 1979); and the second looks at the social impact that a hashtag is able to generate and is represented by hashtag activism.

Regarding the first sub-branch we must remember that the social media like Twitter since they have appeared, have been the object of numerous studies and of various thematic in-depth analyses. There is their theorization as a connective media (van Dijck, 2013), they therefore intervene on the way of defining social bonds through forms of connection that mix social and socio-technical norms typical of online environments (van Dijck, 2013), creating a symbolic field and digital cultural practices that delimit specific ways of relating—often distinct from those offline—and which preside over new processes of signification of being together. Connective media (van Dijck, 2013) have become an almost uninterrupted presence in daily routines: they absorb a significant part of identity processes and social relationships; they give life to a common heritage of cultural and symbolic practices, rules, behavioral practices that contribute to settling “an accepted version of reality” and intersubjectively shared within the same communicative environment.

This is where that line of study comes in that analyzed the dimension of connections between users, identifying the tweets as a tool to provoke reactions in the audience (Marwick and boyd, 2010), and to generate ad hoc publics and that interact between them through the functionality of commenting on posts and proposing them again (retweeting); these effect are attributable to affordances that represent a relational approach to understanding how people interact with technology (Leonardi, 2013). Rathnayake and Suthers (2018) extensively analyzed affordances in digital environments—specifically Twitter—to argue that hashtags are affordances for “momentary connectedness”. According to Rathnayake and Suthers (2018), Twitter hashtags can be seen as affordances for two reasons: (1) the platform allows the creation of hashtags and (2) through hashtags different types of actions emerge. On this trail are placed the studies that analyse hashtags as tools for collective responses (Ross, 2020), expressions of solidarity (e.g., De Cock and Pizarro Pedraza, 2018), support for advocacy strategies (Saxton et al., 2015), strategies of inclusion oriented to create a joint sense of belonging in the community formed around it (Mulyadi and Fitriana, 2018), and strategies to drive television advertising and consequently commercial products (Arvidsson and Caliandro, 2015; Stathopoulou et al., 2017).

To the analysis of hashtag affordances by Rathnayake and Suthers (2018), we add a third reason: (3) the possibility of hashtags to change their original meaning thanks to retweets and quotings, knowing, however, that this happens for topical hashtags, that is, those for which there is an interest from an audience. The flow of conversation that is generated transforms hashtags into public speeches that are assembled through the multiple contributions of users. We can look at this as an evolution of what happened in 2007 with #sandiegofire, allowed by the interaction methods of social media and by the action of users who comment, reply, post messages and images, or other data, actively participating in the evolution of media events or crises.

Thus, there are two types of events that generate a large number of reactions in hashtags: “media events” (from major sports and entertainment broadcasts to election-night political coverage) and “acute events” (from natural disasters to political unrest) (Bruns et al., 2016). In the first case, hashtags linked to official events are used to follow the expression of shared fandom in the context of a major, internationally televised annual media event, as in the case studied by Highfield et al. (2013) for Eurovision Song Contest, where researchers followed and analyzed the evolution of meanings for hashtags: #eurovision, #esc, and #sbseurovision. The second case could be represented by the studies on Queensland floods in 2010-2011 and Christchurch earthquakes (e.g., Bruns and Burgess, 2012, 2014; Bruns et al., 2012) and now for the Covid-19 pandemic (e.g., Boccia Artieri et al., 2021a,b; Kurten and Beullens, 2021; La Rocca and Greco, 2022). In this case, there is a prevailing idea that when crises or disastrous events emerge, hashtags are born with them, as a need felt by social media users to share information, follow news, and comment on them. They do this by using a unique collector, the hashtag, to ensure the flow of information. In this way, discursive assemblages (Rambukkana, 2015) are generated under the hashtags, which are the result of an interconnected network between multiple meanings that are gradually being built and the organizational network made up of media and their features. This process was spotted by Rambukkana (2015), who defines hashtags as generated from “nodes in the becoming of distributed discussions” (Rambukkana, 2015, p. 3). The studies that are placed within this field analyse the content of tweets, working on the analysis of the texts (e.g., Behzadidoost et al., 2021), therefore of the textual traces left by users as comments. Others begin to look at how a network of relationships is generated through hashtag streams (e.g., Pilar et al., 2021), then examine which actors and topics are the subject of public debate.

The second sub-branch appears as a direct consequence of the first, because Twitter hashtags are bottom-up user-proposed tagging conventions that embody user participation in the process of hashtag innovation as it pertains to information organization tasks (Chang, 2010) and, subsequently, the spreading of activism and participation in political and social themes (e.g., Sebeelo, 2021), the creation of counternarratives and counterpublics different from those spread by the mainstream (Jackson and Foucault Welles, 2015). These have also been related to the viral phenomenon of racialised hashtags (e.g., Sharma, 2012; Jackson, 2016), or feminist movements such as #MeToo (Dobrin, 2020) or LGBTQ+ movement (e.g., Duguay, 2016). This is hashtag activism, within which a hashtag becomes a flag under which the activists gather their protests. In these studies, the hashtag becomes the symbol and manifesto of a movement.

These are, therefore, forms of demonstration conveyed by hashtags that embody forms of social disaffection and that find expression within digital platforms (van Dijck et al., 2018), specifically in the Twittersphere. These forms fall within hashtag activism, which refers to demonstrative practices that allow to aid “ordinary people and those without access to traditional forms of power create compelling, unignorable narratives” (Jackson et al., 2020, p. 185).

The narrative agency in hashtag activism was analyzed by Yang (2016), who defined it as generated by a broader online activism, as the outcome of multiple individual actions, by multiple individuals, to link a social or political claim under a common word, phrase, or hashtag. The temporal unfolding of these messages, which are mutually connected in networked spaces, provides the shape and strength of a narrative agency. One can consider the form of discursive protests on social media in the use of #BlackLivesMatter, mostly in the United States, a hashtag that was generated in response to the acquittal of George Zimmerman in July 2013 in the fatal shooting death of African-American teen Trayvon Martin, which produced a protest movement both in the streets and on social media networks.

Recently Sebeelo (2021) working with the hashtags #ThisFlag (Zimbabwe) and #RhodesMustFall (South Africa) highlighted that social media has accentuated resistance in Africa and, although African continent still lags behind in smartphone ownership and internet connectivity compared to other regions of the world, there is enough evidence to suggest that online-based movements have produced a transformation in political engagement in Africa. Agency narrative can also be found in Dobrin's study (2020) on #MeToo, which emerged in response to the many sexual harassment accusations involving high-profile Hollywood producer Harvey Weinstein and the numerous celebrities and others who spoke up about the abuse they experienced at the hands of the film mogul, leading him to be brought to justice. In #MeToo study, digital activism and the hashtag's use are read for their cultural importance, emphasizing the symbolic role played by the hashtag in the emerging myth around the movement through its narrative use, shaped by its users (Dobrin, 2020). These uses demonstrate the “power of digital activism in shaping public discourse” (Yang, 2016, p. 13).

In hashtag studies and hashtag activism, the object of interest is topical hashtags, that is, those capable of generating a conversational flow in the digital environment. Bruns and Stieglitz (2012) argue that hashtags linked to a crisis event, or a television program are more likely to give rise to a sharing of information, which engages and disseminates other information conveyed by URLs, photos, and continuous retweets than hashtags with generic content and meaning (not topical hashtags, i.e. #job, #holidays). This leads Bruns and Burgess (2015, p. 21) to infer that “these distinctly divergent, stable patterns in user activities for these different hashtags use case points to the existence of different conceptualisations by users of the hashtag community that they are seeking to address or participate in”.

Therefore, there is an awareness of the use of tools and a redefinition of operations that can be generated through the hashtag dissemination circuit. This awareness of use manifests through the creation of memory hashtags for particular events to which photos are attached and spread on platforms such as Instagram (Serafinelli, 2020). On Instagram, Twitter, and other platforms, the conscious use of hashtags and features has been established, developing the capacity for collective action.

### Appraisal of the second main stream

This conscious use has generated a set of metrics to define user behavior. Hence, it is possible to divide the second strand into two sub-branches, the one that includes research that uses metrics to describe data flow within social media, as in descriptive statistics; and the other that considers strategies of inferential statistics to study user behaviors and then how to act to influence these behaviors, to satisfy these last purposes, however, it is necessary to identify ad hoc indicators for monitoring hashtags.

Within this second block, a dividing line is represented by the platformization process (Helmond, 2015), that has saturated every area of the web and in which institutional and non-institutional actors move, carrying out a new intermediation function; it is this new intermediation that structures the information and commercial flow through the use of users' behavioral data, subjecting them to the logic of algorithms.

What we are witnessing is, on the one hand, the rise of the platform as the dominant infrastructure and economic model of the web and, on the other, the convergence with social media, as platforms, in building an increasingly integrated ecosystem. To enable this process, the tech companies have operated on dynamics relating to decentralization in data production and re-centralization of data collection, aiming at: (a) making the external data “platform ready,” i.e., suitable for operation in the platform model—for example, with the use of the Facebook “Like” button for content on the web; (b) making internal data useful for third-party development—for example, the increasingly regulated use of API (Application Programming Interface), which allow you to access a portion of the platform data for your own purposes (Boccia Artieri et al., 2021c).

Therefore, the effect of platformization takes on de facto a political nature, since in making external data suitable for the platform, it gains greater control over how the contents appear when they are shared. The power and politics of platforms also extend beyond them, favoring certain protocol logics that adapt external contents to their own internal language (and priorities). Ultimately, we are faced with what Helmond (2015) defines as the “double logic of platformization”:

“This double logic is operationalized through platform-native objects such as APIs, social plugins, and the Open Graph, which connect the infrastructural model of the platform to its economic aims. These elements serve as prime devices for social media platforms to expand into the web and to create data channels—data pours—for collecting and formatting external web data to fit the underlying logic of the platform” (Helmond, 2015, p. 8).

In practice, the platforms provide a technological framework on which others are led to operate and the data produced by others becomes readable by the platforms that can use them in a useful way for their own economic model. Thus, we are witnessing a transition that is not only technological, but also social, economic and political and is based on the new tracking of behavioral data.

In the first period we found a network of descriptive measures for novelty in social media that is represented by topics trending on Twitter or other platforms as expressed by hashtags. These descriptive measures expand rapidly thanks to the functionality of the API, to which the first sub-branch is attached. The APIs allow you to download data-tweets, for example, keeping elements useful for analysis, such as: hashtags, tweets, re-tweets, mentions and other information from which it is possible to reconstruct the meanings and relationship networks. Here we also identify operational indications regarding how to work with tweets, mainly by Bruns and Stieglitz (2013). As scholars suggest (2013, p. 93) it's important to report the values for: the original tweets sent “tweets which are simply original statements, without mentioning other users”; the total number of retweets; and inside the RTs is useful to find the @replies sent, which constitute @mentions sent that are “tweets which contain “@user”, but no indication that the message is a retweet of an earlier post by user” and RT plus @mentions, which represent “tweets which are in the format “RT @user [original message]” or equivalent”. It is also important to indicate the total number of tweets making up the dataset and the presence of URLs that provide an “indication of the amount of external resources a user is introducing into, or retweeting from, the hashtag conversation”; another element is the geolocation.

Bruns and Stieglitz (2013), suggest three areas of metrics in the study of Twitter hashtag datasets: (1) user metrics, which include “metrics about a user's activities, and metrics about their visibility within the overall community of hashtag participants” (p. 97); (2) temporal metrics, which envisage a “a breakdown of the total dataset not by user, but by time” (p. 99); (3) combined metrics, which combine the use of temporal metrics with user and user percentile metrics to identify “differences between the activities of leading user groups and the more random contributions by less active users that have already been identified” (p. 101). The metrics were developed as a requirement in the social research sector and in the economic-business sector.

The use of descriptive measurement tools has established itself in the business sector because they enable companies to learn about their brand reputation or engagement around a product and service. The same is true for various metrics including awareness metrics, which provide information on current and potential audiences; engagement metrics, which show how much and how the public is interacting with the brand; conversion metrics, which measure the effectiveness of social media marketing actions; and customer metrics, which reflect how active customers think and feel about a brand (Shleyner, 2020). These metrics are an attempt to overcome vanity metrics, which as Rogers (2018, p. 450) explains, are “a critical term from business studies that demonizes analysts for a reliance on the brute counting of page views and likes as indicators of success in the hit and like economies (Ries, 2009; Gerlitz and Helmond, 2013)”.

To overcome vanity metrics, Rogers (2018) suggests “critical analytics”, linking this conceptualization, relating to metrics, to the changes in social media, which are no longer the same environment as 5 or 10 years ago. This is the starting point for the second sub-branch of this block of studies, which, as already indicated, marks the transition from social media to digital platforms. Roger considers whether metrics measure or prompt behavior. This question arises because, considering that the number of likes, page views, and shares appear on websites, social profiles, posts, and tweets, we must ask if this score fuels the desire for vanity. It nourishes the desire to achieve fame and success, to be a micro-celebrity, or to treat the audience as a “fan base” (Marwick and boyd, 2011; Senft, 2013). This pushes users to take action to increase the impact of these vanity metrics and, in turn, it feeds the desire to extract more information from “found objects”, things created for another purpose, and we can run into an inconvenience, which is to find ourselves with a lot of behavioral data, without any meaning associated with it (Fielding, 2019).

There are difficulties inherent in “what to measure” on social media to extract “what type of information”. Critical analytics address this difficulty by shifting the axis of interest from the studied vanity networks to the problem network, which emerges through hashtag activism. It is no coincidence that Rogers (2018) reports as an example #BlackLivesMatter on Twitter, where “unity is conceived as hashtag discipline, numbers as recognized contributors, and commitment as repeated participation” (2018, p. 455). Here, he explains social media as one space used to vent social problems. He also highlights that metrics should thus “measure the “otherwise engaged” or modes of engagement (other than those for vanity metrics), such as dominant voice, concern, commitment, positioning, and alignment” (2018, p. 467) in continuity with analytics activism (Karpf, 2017). In the wake of Rogers' work (Rogers, 2013, 2018, 2019), the contribution of Omena et al. (2020), developed from a three-layered (3 L) perspective, emphasizes the need to follow the evolution of the medium, communication technologies, their own methods, and the consequent availability of digital data. The 3 L perspective “serves as a form of “critical analytics” or “alt metrics” for social media research by locating issue networks and creating indicators that are alternatives to marketing-like measures” (Omena et al., 2020, p. 4). From this perspective, hashtag engagement is seen as “collectively formed actions mediated by technical interfaces” (Omena et al., 2020, pp. 4–5). The identified levels are as follows. The first is represented by differences between high visibility and ordinary hashtag usage culture, its related actors, and content. The second focuses on hashtag activity and repurposes hashtags by looking at how they can be embedded differently in social media databases. The third considers the images and texts related to hashtags.

In other words, the engagement of hashtags is described as a “grammaticised” action that moves toward descriptions of images and feelings or toward particular topics of discussion (or issues), which require a (minimum) collective level of commitment (Omena et al., 2020). Omena et al. (2020) developed a multidimensional approach that considers the hashtag an element—a prism—with many faces. However, their approach is at a procedural rather than conceptual level, and techniques are developed.

## Discussion

This study aimed to produce a meta-synthesis of the existing studies on hashtag research [RQ], to highlight what has been overlooked and what new avenues can be examined to advance knowledge. Research using hashtags is a composite field of study, which required to adapt the classic meta-study procedures on it. To organize the salient elements that emerged from literature review, we can use some of the procedures identified there (Ronkainen et al., 2022). Thus, what emerged for the two lines of studies can be divided into the sub-branches of: theoretical perspectives and the social media (meta-theory); type of study (meta-method); analysis of results or conclusions (meta-data analysis) (see Table 1).

**Table 1:** Summary of the results of the meta-study.

		**Key findings: meta-theory**	**Key findings: meta-method**	**Key findings: meta-data-analysis**
First block	Hashtag studies	The media are identified as connective media and affordances are the conceptual framework that is most used to explain the actions of users especially on twitter	These are theoretical studies whose main objective is to theorize the changes in the meaning and use of hashtags	The main results consist in having identified an awareness of the use of hashtags by users within social media
	Hashtag activism	The Twittersphere enters the identification processes of digital platforms. Through the use of hashtags, those who do not have access to traditional forms of power have the ability to create compelling narratives that cannot be ignored outside digital environments. Online and offline merge within the platform society	These are theoretical and empirical studies that underline the role of hashtags as demonstrative forms of protest	The hashtag becomes a flag, the symbol and manifesto of a movement
Second block	Descriptive measures	The first operational indications are drawn on how to work with tweets considering its features	These are studies on metrics called vanity metrics	They are identified different areas of metrics in the study of twitter hashtag datasets: user metrics, temporal metrics, combined metrics
	Behavioral data	The connection process undergoes a metamorphosis, and we enter in the platformization. In addition to twitter, instagram is also studied	The metrics now become “critical analytics”, linking this conceptualization, relating to metrics, to the changes in social media, which are no longer the same environment as 5 or 10 years ago	Difficulties emerge with respect to “what to measure” on social media to extract “what type of information”. The works on alt-metrics and that of 3 l perspective that serves as a form of “critical analytics” for social media research by locating issue networks and creating indicators that are alternatives to marketing-like measures

In Table 1, among the two blocks, the first represents the thematization of the role of the hashtag and the second, the search for a measurement of its impact. Both blocks were confronted with the techno-social changes that occurred over the years. Consequently, three orders of considerations can be produced.

The first consideration concerns the way in which scholars have viewed the hashtag within the two blocks. In the first stream of research on hashtag and activism hashtag, studies treated hashtags as social entities conveyed first from connective media and then from digital platforms. Following the technological changes of the web infrastructure, which moved from Internet-based to Web 2.0, and then to digital platforms, hashtags have similarly undergone a change in their spreadability (Jenkins et al., 2013). These structural changes reflect the evolution of the source/environment characteristics (see Table 2).

**Table 2:** The dimensions of hashtag analysis.

	**Sense-making practice**	**Propagation-effect practice**
Source	Connective media	Digital platforms
Content	Social entity	Tool entity for buzz or engagement
Process	Reconstruction of sense-making	Reconstruction of interest: buzz, engagement

In hashtag studies and activism, the hashtag is treated as a unit of meaning formed through a process of attribution of contributions/comments inserted by each user within the movement's label. Thus, through the analysis, a researcher examining this hashtag category can reconstruct the meaning and sense attributed by others to what the hashtag represents. From this perspective, hashtag is a sensemaking practice (see Table 2). Here, a gap in the literature becomes apparent; that is, the reconstruction of the meaning of a hashtag—a “before” and an “after”, represented by the literal meaning of the label/event, and by its modification through reworking by users, respectively—is not considered.

In the second stream of studies, the hashtag became a tool for estimating the impact of and interest in an event or theme. Here, digital platforms represent the source/environment in which the hashtags spread. The hashtag is treated as a unit of sensemaking, formed through the generated buzz or level of engagement. Therefore, it becomes the effect produced by the interaction of many people. Through the analysis, a researcher can reconstruct the volume of interaction between users regarding a theme or an event of interest (see Table 2), estimating the propagation effect. For example, similar to a sound wave, a hashtag also spreads across platforms, with an alternation of compressions and rarefactions. These are detected by a receiver, such as a user, social researcher, and media outlet, as changes in pressure. Specifically, this propagation is estimated within the platforms. Here, a second lack emerges: if social media platforms are digital structures, and if the distinction between online and offline has collapsed, it is necessary to estimate the presence/absence of the propagation effect inside and outside digital environments.

The second consideration is closely related to the first and has already been introduced as a shortcoming. It is concerning time and the interchange between social environments. Time comes into play when analyzing the development of extra and collateral meanings in the hashtag; consequently, we must deal with a before and an after. Time is also a social factor, a period in which user interaction develops. The importance of the time factor appears in the theoretical contribution of Faltesek (2015), who links the temporal question not only to the collection and interpretation of hashtags, but also to the historical-social time of the event that generated them. That is, instead of viewing the issue strictly through digital and research functions on social platforms, he anchors them to a cultural and social dimension. He begins by distinguishing kairos—“a time in between”, an indefinite period of time in which “something” special happens—and chronos, which refers to chronological and sequential time. In social media, kairos cannot be determined at the individual level represented by the posts (read or unread), or by the elements attached to hashtags, enabled by polysemic digital communication. Rather, it must be sought in the temporal (and social) context within which hashtag networks are generated and within which they are located. They operate by activating an audience, known as ad hoc publics (Bruns and Burgess, 2015), which follows a specific hashtag, shares it, and uses it in combination with other hashtags. Sharing produces a clamor, which can be linked exclusively to the digital environment or can come out of it; however, it can also enter it by virtue of something that has happened outside. Thus, it is important to consider how, where and when this propagation effect develops.

To estimate the timeframe, one can consider media hype the starting point. Vasterman (2004, 2005) explored this concept, defining it as “a media-generated, wall-to-wall news wave, triggered by one specific event and enlarged by the self-reinforcing processes within the news production of the media. During a media hype, the sharp rise in news stories is the result of making news, instead of reporting news events, and covering media-triggered social responses, instead of reporting developments that would have taken place without media interference” (2018, p. 20). Therefore, it is necessary to consider when the event contained in the hashtags began to be of interest to media outlets; consequently, it is necessary to estimate its coverage in the press, for example by identifying when it appears for the first time, how quickly it grows every day, and when it stops being of interest to the news media. This provides an overview of the life cycle of the news and the evolution of users' interest. In addition to the media hype, it is necessary to check the number of tweets and posts that are produced in the same period, and examine if there is a correspondence between the two. According to Pang (2013), social media hype develops as a “netizen-generated hype that causes huge interest that is triggered by a key event and sustained by a self-reinforcing quality in its ability for users to engage in conversation” (p. 333). The introduction of these elements—of time or period, of storm or engagement, of interdependence or lack of relationship between media and social media hype—builds a framework through which to interpret of the research results.

**Figure 1:**
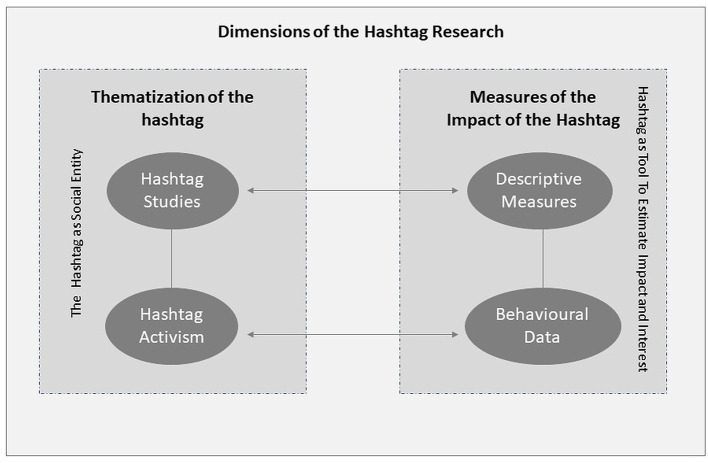
Current dimensions of the hashtag research.

The third question concerns the emergence of nascent hashtag research based on the elements identified here. We can combine the sub-branches of the two main strands and obtain the conceptual space within which the current hashtag research moves (see Figure 1).

We identified two main branches of studies represented by studies, in which we deal with: 1) thematizing the hashtag. They are contained in the hashtag studies and hashtag activism labels, in which the hashtag is considered a social entity; 2) measuring the effects of hashtags. These are represented by the search for measures and parameters to describe and analyse them and subsequently, to identify behavioral data through the metrics connected to them. We can also consider how the respective sub-branches are connected to each other vertically and horizontally.

Along the vertical dimension of the hashtag as a social entity, hashtag studies are an essential prerequisite for the development of hashtag activism. Indeed, if hashtag is not viewed first as a subject capable of changing its meaning through the use and interaction between users, it cannot be considered a flag, a manifesto within which the social instances are collected. Conversely, hashtag as a tool to measure interest and impact testifies to the need to identify parameters for the description of its use, diffusion within social media and the effects that its spreadability are able to produce. Therefore, here, the indications that emerge from the appraisal of the studies suggest that all useful elements are identified to describe the volume and methods of dissemination, and subsequently—due to the transition from connective media to digital platforms—the need to identify metrics emerges, more specifically able to read user behavior.

Along the horizontal axis—which in Figure 1 is indicated by a bidirectional arrow—we identify the links between the conceptual dimension of the hashtag and its articulation in measures. There is a close link between how the hashtag is conceptualized and the development of relevant measurement tools. The hashtag as an entity that changes the meaning from post to post should explain how the space for discussion and sharing within which it moves is organized. Bruns and Stieglitz (2013) describe the features of Twitter and how to work with them, and simultaneously, the two scholars develop metrics to view who uses the hashtags, the organization of the datasets along a time dimension and the possibility of combining both metrics. Along the second horizontal axis we find the need to look at hashtags as social behaviors, which find their strength in being used by the community to express forms of protest. Here the alt-metrics of Rogers (2018) emerge and are inserted.

At this point we can argue that we have answered our research question, whether it is possible to outline the traits of the nascent hashtag research. However, we have focused on meta-synthesis to do this, as it offers the possibility of revealing new insights, if it is possible to trace new spaces for this area.

Two different ways of looking at hashtags emerged from the appraisal of the studies (see Table 2), which are a result of our research, since they are not explicit in the analyzed studies. Furthermore, we understand that the work on hashtags, on the texts connected to them, on the analysis of the relationships generated through them or on the metrics, is a post-reconstruction work conducted by the researcher. Whether it is a social entity or propagation-effect practice, the researcher's work consists of a reconstruction operation, which differs from studies with surveys or interviews as there is no question stimulus. The researcher does not direct the stimulus, which, in this case, is a hashtag. The stimulus for discussion, response to the discussion, and interest in the topic are all compressed in the hashtag. Therefore, specifying how the hashtag is viewed or framed in the research design is necessary in the conceptualization process and consequently, to explain which measurement parameters are adopted and for what reasons. Furthermore, the effect of propagation and interconnection between the different social structures within which the instance/theme/event that the hashtag represents spreads, must be considered. Additionally, in this case, the researchers should specify the reasons, because they follow the hashtag only within a media environment or because they consider the interconnection in the analysis of the hashtags of the media ecosystem.

These procedural and a priori indications that can transform research with hashtags from a simple description of a social phenomenon into theories focused on a given object of study, by putting forward a series of assumptions to derive and empirically verify specific hypotheses over time because a hidden dimension of studies is made explicit and made comparable in other works of the same type. This allows you to compare the results, because a parameter is indicated that can be taken as a reference by various scholars. And it specifies what conceptual-procedural strategy is being followed in order to move from a mass of data without structure to an interpretation.

**Figure 2:**
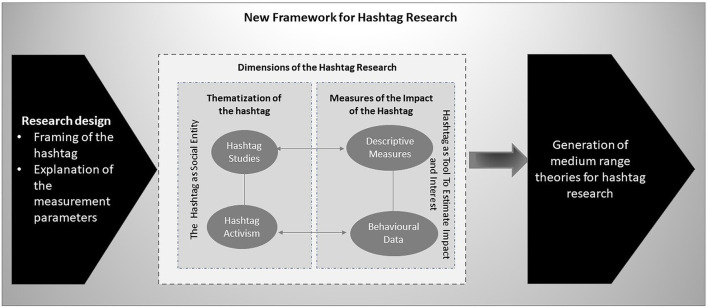
New perspectives for hashtag research.

The integration of these elements into the hashtag research is able to outline future developments for what is becoming a specific research sector (see Figure 2).

In this way, future directions for hashtag research are outlined, which go toward a sedimentation of the results and the construction of a research area.

## Conclusion

In an era in which research is data-driven and the focus is on the visualization of big data, addressing issues related to the conceptualization of the object of analysis may seem unpopular. However, the elements we propose here to add to the studies that fall within the hashtag research offer some advantages.

First, they clarify the orientation of the research, allowing the contextualization of both through framing of the hashtag in the study, and in data collection and in analysis of the results. Second, they are applicable to all fields in which the study of hashtags is widespread. They also pave the way for combined studies along the axes—horizontal and vertical—of the dimensions of the research hashtag. In the face of this, there are also some limitations that this study has. Some of these limits are inherent in the meta-synthesis which is often accused of being reductionist; and another is the partiality of the studies selected to bring out the evidence discussed in this study. Compared to the limitations inherent in the meta-synthesis, we follow the approach of Walsh and Downe (2004, p. 205) who maintain “In response to the postmodernist critique that synthesis is reductionist, it may be helpful to view the process as opening up spaces for new insights and understandings to emerge, rather than one in which totalizing concepts are valued over richness and thickness of description”. And it is precisely by following this characteristic of meta-synthesis that we have used it here. Regarding the selection of the studies, we specified that it followed the berrypeacking model, however most of the studies found and discussed here deal with Twitter and Instagram, leaving out the other platforms.

However, we consider the impact of this element in our study to be negligible because the indications added to the hashtag research (see Figure 2) are placed upstream of the choice of the platform on which to work.

Another element to consider is the transience of this study due to the evolution of technologies and related social dynamics. This study is a reflection of today and we are unsure of whether it will remain valid tomorrow. However, it is nevertheless a starting point.

## Author contributions

These authors contributed equally to this work. All authors contributed to the article and approved the submitted version.

## Conflict of interest

The authors declare that the research was conducted in the absence of any commercial or financial relationships that could be construed as a potential conflict of interest.

## Publisher's note

All claims expressed in this article are solely those of the authors and do not necessarily represent those of their affiliated organizations, or those of the publisher, the editors and the reviewers. Any product that may be evaluated in this article, or claim that may be made by its manufacturer, is not guaranteed or endorsed by the publisher.
